# Effect of tongue‐hold swallow on posterior pharyngeal wall using dynamic area detector computed tomography

**DOI:** 10.1111/joor.13246

**Published:** 2021-09-28

**Authors:** Keiko Aihara, Yoko Inamoto, Daisuke Kanamori, Marlís González‐Fernández, Seiko Shibata, Hitoshi Kagaya, Satoshi Hirano, Hiroko Kobayashi, Naoko Fujii, Eiichi Saitoh

**Affiliations:** ^1^ Faculty of Rehabilitation School of Health Sciences Fujita Health University Toyoake Japan; ^2^ Department of Rehabilitation Medicine I School of Medicine Fujita Health University Toyoake Japan; ^3^ Department of Density and Oral‐Maxillofacial Surgery School of Medicine Fujita Health University Toyoake Japan; ^4^ Department of Physical Medicine and Rehabilitation Johns Hopkins University School of Medicine Baltimore Maryland USA; ^5^ Department of Rehabilitation Fujita Health University Hospital Toyoake Japan; ^6^ Department of Radiology School of Medicine Fujita Health University Toyoake Japan

**Keywords:** dysphagia, multidetector computed tomography, pharynx, swallowing, tongue, tongue‐hold swallow

## Abstract

**Purpose:**

The purpose of this study was to elucidate the effects of the tongue‐hold swallow (THS) on the pharyngeal wall by quantifying posterior pharyngeal wall (PPW) anterior bulge during the THS. In addition, the effect of tongue protrusion length on the extent of pharyngeal wall anterior bulge was analysed.

**Methods:**

Thirteen healthy subjects (6 males and 7 females, 23–43 years) underwent 320‐row area detector CT during saliva swallow (SS) and THS at two tongue protrusion lengths (THS1 protrude the tongue as much as 1/3 of premeasured maximum tongue protrusion length (MTP‐L) and THS2 protrude the tongue as much as 2/3 of MTP‐L). To acquire images of the pharynx at rest, single‐phase volume scanning was performed three times during usual breathing with no tongue protrusion (rest), protrusion of the tongue at 1/3 of MTP‐L (rTHS1) and protrusion of the tongue at 2/3 of MTP‐L (rTHS2). Length from cervical spine to PPW (PPW‐AP) and the volume of pharyngeal cavity was measured and was compared between rest, rTHS1 and rTHS2 and between SS, THS1 and THS2. Correlation between MTP‐L and PPW‐AP was calculated in three conditions, SS, THS1 and THS2.

**Results:**

PPW‐AP at rest, rTHS1 and rTHS2 was 2.9 ± 0.6 mm, 3.0 ± 0.5 mm and 3.0 ± 0.5 mm, respectively, showing no significant differences across swallows. PPW‐AP at the maximum pharyngeal constriction was 8.1 ± 2.0 mm, 9.1 ± 2.4 mm and 8.7 ± 2.0 mm in SS, THS1 and THS2, respectively. Compared to SS, PPW‐AP in THS1 was significantly larger (*p *= 0.04) and PPW‐AP in THS2 was not significantly different (*p *= 0.09). Pharyngeal volume at rest, rTHS1 and rTHS2 was 16.4 ± 5.2 mm^3^, 18.4 ± 4.5 mm^3^ and 21.3 ± 6.2 mm^3^, respectively. It was significantly larger during rTHS2 compared with rest or rTHS1 (rTHS2‐rest *p *= 0.007, rTHS2‐rTHS1 *p *= 0.007). Pharyngeal volume was completely obliterated (zero volume) at maximum pharyngeal contraction in all except one subject. There was no correlation between MTP‐L and PPW‐AP in any of the three conditions (SS, THS1 and THS2).

**Discussion:**

This study demonstrated that the expanded pharyngeal cavity due to the tongue protrusion was completely obliterated by the increase in anterior motion of pharyngeal wall during THS. It also became clear that the degree of tongue protrusion did not linearly correlate with the movement of PPW during THS. There was no relationship between PPW motion and the MTP‐L, suggesting that the effect of tongue protrusion is better determined in each subject by analysing the motion of PPW using imaging tools.

## 1 INTRODUCTION

The tongue‐hold swallow (THS) is a swallowing exercise performed by protruding the tongue maximally and holding it between the central incisors during saliva swallows to strengthen pharyngeal contraction.^1^ THS was originally developed after observing deviated pharyngeal wall movement in some patients. Fujiu‐Kurachi et al. reported increased posterior pharyngeal wall (PPW) anterior bulging during swallowing in postoperative tongue cancer patients whose anterior tongue was resected.^2^ They suggested the change in PPW movement was compensatory as there was reduced base of tongue movement (BOT) due to disturbed tongue motion anchoring.^2^ Their subsequent study in healthy subjects, comparing the liquid swallow with no maneuver and with the tongue holding maneuver, confirmed that THS reduced BOT movement range and increased the range of the PPW. They suggested that restraining the motion of BOT by protruding and holding the tongue during swallowing had the potential to increase PPW to compensate.^1^ Based on these two studies, the THS has been used for the treatment of patients with dysphagia who have reduced contact of PPW and BOT to facilitate pharyngeal contraction during swallowing.^1^ However, the physiologic changes induced by the THS and the appropriate method to perform the THS have not been fully understood due to the lack of kinematic data.

The effects of the THS have been measured using manometry, tongue pressure, electromyography and surface electromyography. Lazarus et al. performed videofluoroscopy (VF) and manometry in three patients with postoperative head and neck cancer and reported that THS increased the contact pressure and duration between the BOT and PPW.^3^ Discrepancies exist in studies using high‐resolution manometry; two reported increased pharyngeal pressure,^4^, ^5^ two reported decrease in pharyngeal pressure,^6^, ^7^ and one reported no change.^8^ Hammer et al. studied the THS using manometry and electromyography and reported that pharyngeal pressure was unchanged but the muscle activity of superior pharyngeal constrictor was significantly increased during the THS compared with saliva swallows.^8^ Nonetheless, they recommended evaluation by direct observation of the PPW to clarify the increase in superior pharyngeal constrictor activity was brought by the increase in anterior bulge of PPW.^8^

One of the difficulties to study the THS kinematically is the visualisation of the PPW during THS. VF allows kinematic analyses, but without contrast, it is difficult to observe the soft tissues when they are contact each other, such as the BOT and PPW during swallowing. THS is basically performed during saliva swallows, and therefore, the PPW position is difficult to observe when it contacts the BOT during VF. To our knowledge, no study of PPW movement has been performed using VF since the aforementioned studies by Fujiu.^2^, ^3^

A recent study using 320‐row area detector computed tomography (320‐ADCT) was performed to study the effect of the THS on pharyngeal constriction three‐dimensionally.^9^ Pharyngeal cavity volume was measured during the THS and compared to the volume during saliva swallows.^9^ Although this study expanded the three‐dimensional pharyngeal dynamic evaluation, difficulties in visualising the boundary between the PPW and BOT made it difficult to analyse the effect of THS on PPW. To overcome this, Kanamori et al. developed a technique coating the tongue surface to visualise the soft tissue using 320‐ADCT.^10^ By using this methodology, it is possible to visualise pharyngeal wall motion even during swallows without contrast.

The importance of adjusting the length of tongue protrusion during the THS has also been studied.^11^, ^12^ Fujiwara et al. reported that 32 mm of maximum tongue protrusion was the cut‐off value to determine the increase or decrease in the tongue pressure during THS.^11^ Oh reported that longer tongue protrusion increased submandibular muscle activity where the tongue was protruded as much as one‐third of the premeasured maximum tongue protrusion length or where the tongue was protruded as much as two‐thirds of maximum tongue protrusion length and THS with maximum tongue protrusion.^12^ These studies suggested that setting appropriate tongue protrusion length during THS is needed for reliable effects. However, these mainly studied the effect on the tongue on suprahyoid muscles, and the effect of tongue protrusion length on PPW was not studied.

In this study, we first aimed to elucidate the effect of THS on the PPW by quantifying the movement of the PPW during THS using 320‐ADCT. Second, we aimed to clarify the effect of tongue protrusion length on the range of pharyngeal wall movement. We hypothesised that (1) the THS would increase the range of PPW movement, compared to saliva swallows and (2) the range of PPW movement would increase as the length of tongue protrusion was increased.

## 2 METHODS

### 2.1 Subjects

Thirteen healthy volunteers (6 males and 7 females, 23–43 years) without prior history of dysphagia were recruited. All subjects provided informed consent for participation after thorough explanation of the purpose, procedures and the risk of radiation exposure. The study was approved by the Institutional Review Board at our university (HM16‐135).

### 2.2 Data collection

#### 2.2.1 Measurement of maximum tongue protrusion length (MTP‐L) and instructions during THS

**FIGURE 1:**
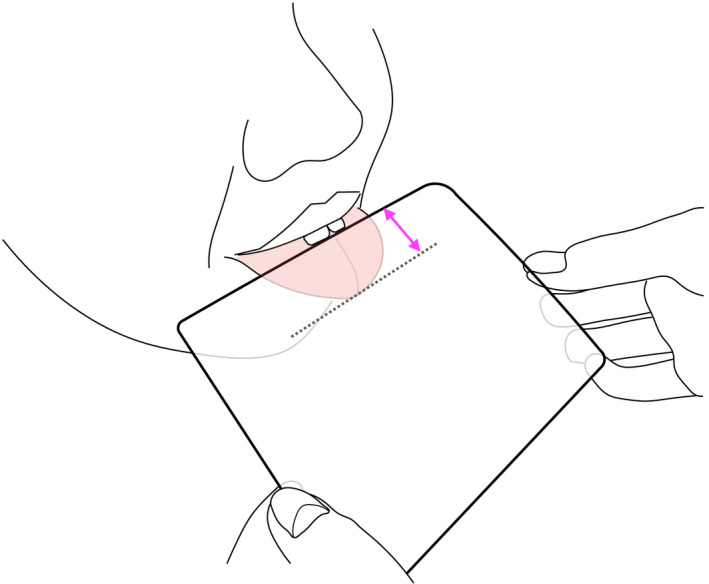
Measurement of maximum tongue protrusion length (MTP‐L). Subjects were instructed to protrude the tongue as much as possible and to hold the protruded tongue with their front teeth. After the transparent film was put on the protruded part of the tongue, two points, the tongue tip and the front teeth, were marked and the distance between the two points was measured as MTP‐L

Subjects were asked to protrude the tongue as much as possible and to hold the protruded tongue with their front teeth. A transparent film was placed on the protruded tongue where the tongue tip and front teeth were marked and the distance between the two points was measured (Figure 1). The subject was asked to protrude the tongue three times, and MTP‐L was defined as the mean of the three measurements. One‐third of MTP‐L (THS1) and two‐thirds of MTP‐L (THS2) were calculated. Subjects were instructed to perform the THS1 and THS2 by the Speech‐language‐hearing therapist. They were instructed to practice the THS1 and THS2 10 times a day for 5 days before the CT imaging.

#### 2.2.2 CT Imaging

Immediately before the CT scanning, the tongue surface was coated with 5% barium mixed sodium alginate solution (Alloid G, Kaigen Pharma Co., Ltd., Hiroshima, Japan), following the method described by Kanamori et al. in order to visually distinguish the tongue base and the PPW. A 320‐row area detector CT scanner (320‐ADCT, Aquilion ONE vision; Canon Medical Systems, Otawara, Japan) was used for imaging. The gantry was tilted 30°, and the CT chair (eMedical Tokyo, Chuo‐ku, Japan; Tomei Brace, Seto, Japan) was positioned on the opposite side of the CT table. The participants were seated on the chair in a reclining position at an angle of 45°. Single‐phase volume scanning and multiphase volume scanning were performed. Single‐phase volume scanning was performed three times while subjects were relaxed breathing as usual (at rest) under the following conditions: (1) no tongue protrusion, (2) protrusion of the tongue at one‐third of MTP‐L (rTHS1) and (3) protrusion of the tongue at two‐thirds of MTP‐L (rTHS2). Multiphase volume scanning was performed during (1) saliva swallows (SS), (2) THS1 and (3) THS2 with the examiner providing verbal cues. The scanning parameters were set as follows: scanning duration 0.275 s for single‐phase volume scanning, 3.3 s for multiphase volume scanning, field of view = 240 mm, tube voltage/current = single phase 120 kV/30 mA and multiphase 120 kV/40 mA. Scanning range was set 160mm from the base of skull to upper oesophagus. Radiation dose has been estimated as 3.24 mSv for three swallows.^13^

Multiphase volume scanning images were reconstructed in 33 phases at 0.1‐s intervals (10 phase/s) by a half‐reconstruction technique.^14^ Multiplanar reconstruction images and 3D‐CT images were created using the scanner's software. The air column at the oral cavity, pharynx and larynx was visualised with a window level of <300 HU, and the hyoid and cranial bones were visualised with a window level of >350 HU.

### 2.3 Data analysis

**FIGURE 2:**
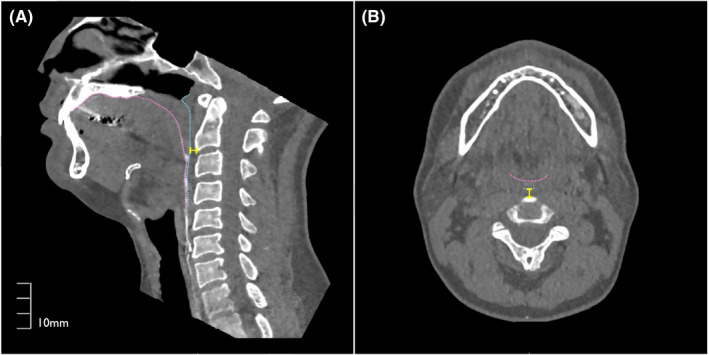
Measurement of the length from cervical spine to posterior pharyngeal wall (PPW‐AP). After rotating the mid‐sagittal images vertically aligning the anterior‐inferior corner of C2 vertebra and the anterior‐superior corner of the C4 vertebra (A), anterior bulge of PPW was identified on the axial cross section (B). The pink dot line shows the surface of the tongue. Blue dot line shows the surface of the posterior pharyngeal wall. Yellow line shows PPW‐AP

**FIGURE 3:**
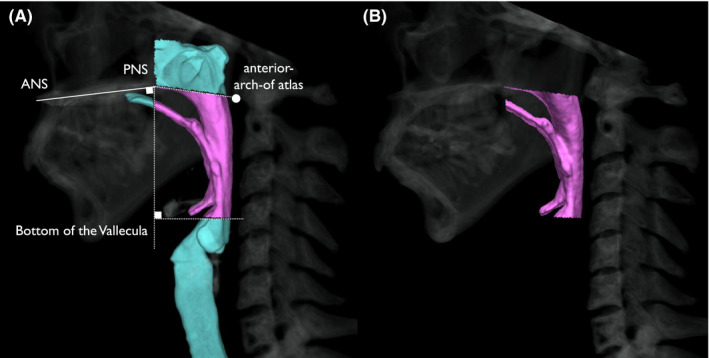
Definition of pharyngeal cavity. (A) Definition of pharyngeal cavity. Superior: the plane through the anterior nasal spine (ANS) and the posterior nasal spine (PNS), parallel to the infraorbital line. Diagonal: the plane inclined at an angle to the superior plane and passing through the inferior border of anterior arch of atlas (C1). Anterior: the plane perpendicular to the top plane and passes through PNS. Inferior: the plane through the bottom of the vallecula, parallel to the superior plane. (B) Depicted 3D‐CT image of pharyngeal cavity according to the definition

The anterior‐posterior length from cervical spine to posterior pharyngeal wall (PPW‐AP) and the volume of the pharyngeal cavity were measured at the three breathing conditions (rest, rTHS1 and rTHS2) and at the maximum pharyngeal contraction during three swallows (SS, THS1 and THS2). PPW‐AP was defined as the length between cervical spine and posterior pharyngeal wall at the height of the anterior‐inferior corner of C2 vertebra. PPW was identified and measured on the axial image after rotating the images vertically aligning the anterior‐inferior corner of C2 vertebra and the anterior‐superior corner of the C4 vertebra (Figure 2). The volume of the pharyngeal cavity was measured using the 3D‐CT images as described by previous study.^9^ The pharyngeal cavity was defined as follows: (1) superior plane—is the plane through the anterior nasal spine (ANS) and the posterior nasal spine (PNS), parallel to the infraorbital line; (2) diagonal plane—is the plane inclined at an angle to the superior plane and passing through the inferior border of anterior arch of atlas (C1); (3) anterior plane—is the plane perpendicular to the top plane and passes through PNS; and (4) inferior plane—is the plane through the bottom of the vallecula, parallel to the superior plane (Figure 3).

To determine interrater reliability, a second rater independently measured forty‐six per cent of the full data set (6 subjects out of 13 subjects).

### 2.4 Statistical analysis

Wilcoxon rank‐sum test was used for the comparison of PPW and volume of pharyngeal cavity across rest, rTHS1 and rTHS2 and across SS, THS1 and THS2. Reliability was tested using intra‐class correlation coefficient (ICC). All the statistical analyses were performed with SPSS Statistics ver. 23.0 (SPSS Japan, Inc). Statistical significance was set at *p *< 0.05.

## 3 RESULTS

All volunteers started SS, THS1 and THS2 without difficulty following the examiner's cue and could finish each swallow within the scan time.

### 3.1 Maximum tongue protrusion length (MTP‐L)

The average MTP‐L was 31.8 ± 6.6 mm. The average THS1 was calculated 10.6 ± 2.2 mm, and THS2 was calculated 21.2 ± 4.4 mm.

### 3.2 Antero‐posterior length from cervical spine to posterior pharyngeal wall (PPW‐AP)

**FIGURE 4:**
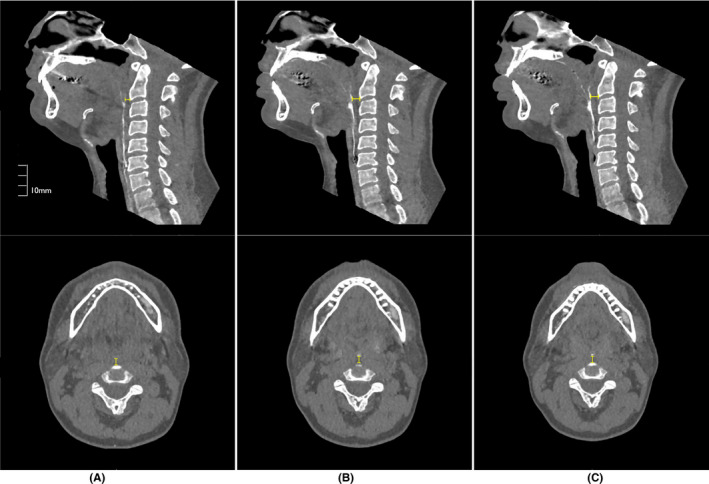
MPR images of one representative subject's anterior bulge of posterior pharyngeal wall (PPW). Upper: mid‐sagittal section, lower: axial cross section at the level of anterior bulge of PPW. (A) SS, (B) THS1, (C) THS2. PPW‐AP was 8.3 mm in SS, 10.3 mm in THS1 and 10.3 mm in THS2

**FIGURE 5:**
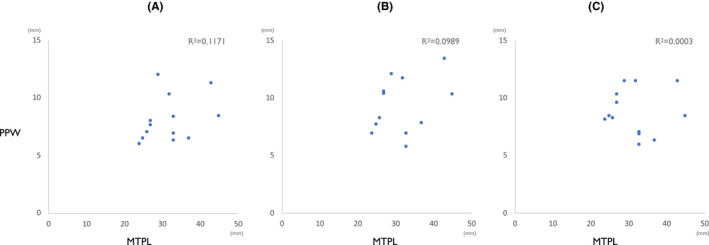
Correlation between maximum tongue protrusion length (MTP‐L) and length from cervical spine to posterior pharyngeal wall (PPW‐AP). (A) SS, (B) THS1 and (C) THS2. The coefficient of determination is shown in the upper right of each graph. There was no correlation between MTP‐L and PPW‐AP in any conditions

Figure 4 showed the measurements for one subject. The average PPW‐AP during the relaxed breathing was 2.9 ± 0.6 mm, 3.0 ± 0.5 mm and 3.0 ± 0.5 mm at rest, rTHS1 and rTHS2, respectively, with no significant difference across conditions (rest‐rTHS1 *p *= 0.179, rest‐rTHS2 *p *= 0.172, rTHS1‐rTHS2 *p *= 0.427) (Table 1). The average PPW‐AP at the maximum pharyngeal constriction was 8.1 ± 2.0 mm, 9.1 ± 2.4 mm and 8.7 ± 2.0 mm in SS, THS1 and THS2, respectively (Table 1). Compared to SS, PPW‐AP in THS1 was significantly larger (*p *= 0.04). PPW‐AP in THS2 tended to be larger, but this difference was not statistically significant (*p *= 0.09). Out of thirteen subjects, nine showed the highest PPW‐AP in THS1, two showed the highest PPW‐AP in THS2, and two showed the highest PPW in SS. There was no correlation between MPT‐L and PPW‐AP (THS1 *R* ^2^ = 0.0989, THS2 *R* ^2^ = 0.0003) (Figure 5).

**TABLE 1:** Results and statistical analysis of PPW‐AP and pharyngeal volume

		Mean (SD)			*P*‐value		
		Rest	rTHS1	rTHS2	Rest‐rTHS1	Rest‐rTHS2	rTHS1‐rTHS2
PPW‐AP	mm	2.90 (0.6)	3.00 (0.5)	3.00 (0.5)	0.179	0.172	0.427
Pharyngeal volume	mm^3^	16.40 (5.2)	18.40 (4.5)	21.30 (6.2)	0.060	0.007*	0.007*

		SS	THS1	THS2	SS‐THS1	SS‐THS2	THS1‐THS2
PPW‐AP	mm	8.10 (2.0)	9.10 (2.4)	8.70 (2.0)	0.039*	0.092	0.196
Pharyngeal volume	mm^3^	0.00 (0)	0.01 (0)	0.02 (0.1)	0.317	0.317	0.317

Note

Value of PPW‐AP and pharyngeal volume as mean and SD (standard deviation) for 13 healthy subjects.

PPW‐AP: length between cervical spine and posterior pharyngeal wall.

Rest: no tongue protrusion during relaxed breathing condition.

rTHS1: protrusion of tongue at one‐third of maximum tongue protrusion length during relaxed breathing condition.

rTHS2: protrusion of tongue at two‐third of maximum tongue protrusion length during relaxed breathing condition.

SS: saliva swallow.

THS1: tongue‐hold swallow with protrusion of tongue at one‐third of maximum tongue protrusion length.

THS2: tongue‐hold swallow with protrusion of tongue at two‐thirds of maximum tongue protrusion length.

* *p *< 0.05.

### 3.3 Pharyngeal volume

The average pharyngeal volumes during relaxed breathing were 16.4 ± 5.2 mm^3^, 18.4 ± 4.5 mm^3^ and 21.3 ± 6.2 mm^3^ for rest, rTHS1 and rTHS2, respectively (Table 1). It was significantly larger during rTHS2 compared with rest or rTHS1 (rTHS2‐rest *p *= 0.007, rTHS2‐rTHS1 *p *= 0.007) (Table 1). rTHS1 volume tended to be greater than rest, but this difference was not statistically significant (*p *= 0.06) (Table 1). The pharyngeal volume at maximum pharyngeal contraction was zero in all except one volunteer who showed complete obliteration in SS, but showed 0.1 mm^3^ in THS1 and 0.3 mm^3^ in THS 2. The PPW‐AP of this volunteer was 6.5, 7.8 and 6.3 mm in SS, THS1 and THS2, respectively.

Two volunteers had smaller PPW‐AP in THS than in SS, but the pharyngeal volume at maximum contractions was zero for all three swallows (complete obliteration of the pharyngeal cavity).

### 3.4 Inter‐rater reliability of the measurements

Intra‐class correlation coefficient (ICC) of PPW‐AP during relaxed breathing/at maximum pharyngeal contraction was 0.985/0.999, 0.973/0.986 and 0.992/0.991 in rest/SS, rTHS1/THS1 and rTHS2/THS2, respectively. ICC of pharyngeal volume was 0.995, 0.993 and 0.995 in rest, rTHS1 and rTHS2, respectively.

## 4 DISCUSSION

It is difficult to observe pharyngeal motion directly during the THS using videofluoroscopy; thus, THS has been investigated mainly by manometry, EMG and measurement of tongue pressure. Those studies identified the effect of THS on swallowing; however, the main effect on pharynx by THS was not described. In this study, the motion of pharyngeal wall during THS was visualised and successfully quantified. The effect of the degree of protrusion on the pharyngeal wall could be also identified.

In this study, 85% of healthy volunteers showed larger PPW‐AP during either THS1 or THS2, compared to SS. The volume of pharyngeal cavity was increased as the length of tongue protrusion increased at rest before swallowing. However, it was completely obliterated in all but one subject during swallowing, regardless of the degree of tongue protrusion. These results suggest that pharyngeal wall contraction increased by increasing the anterior bulge of the PPW to obliterate the expanded pharyngeal cavity. Fujiu & Logemann reported the involvement of the glossopharyngeal portion of the superior constrictor muscle during THS.^1^ Hammer et al. reported increased amplitude of the superior pharyngeal constrictor muscle during THS.^7^ Our study showed increased PPW anterior bulge during THS, supporting that THS increases the anterior movement of the pharyngeal wall by increasing the muscle activity of superior pharyngeal muscles.

Two subjects did not show increased PPW‐AP during THS. Nonetheless, they had complete pharyngeal contraction. It is assumed these subjects completed pharyngeal contraction by increasing tongue base retraction. Previous reports using tongue pressure and sEMG discussed the involvement of tongue and superior hyoid muscles during THS.^11^, ^12^ We speculate that in spite of the suppression of tongue retraction due to the anchoring of protruded tongue, some subjects can still retract the tongue to obliterate the pharyngeal cavity during THS. For these subjects, THS may work for strengthening tongue muscles. Further analysis is necessary to clarify the effect of tongue base motion during THS. It also became clear that subjects might use different strategies to perform THS.

Our second hypothesis, that longer tongue protrusion resulted in greater range of pharyngeal wall movement, was rejected. Only two subjects showed the greater PPW‐AP in THS2, but most showed the greater PPW‐AP in THS1. The degree of tongue protrusion did not linearly correlate with the movement of PPW during THS in some subjects. The load of 2/3 protrusion of MTP‐L was considered too heavy to elicit the PPW anterior bulge for some subjects, suggesting the adjustment of appropriate length of tongue protrusion is essential to achieve the desired effect during THS.

These results are consistent with the previous study reporting that tongue pressure was increased in 11 subjects at THS with greater tongue protrusion, but was decreased in 7 subjects.^11^ They reported that the individual's MTP‐L determined the increase or decrease in tongue pressure at THS with greater tongue protrusion.^11^ If the individual's MTP‐L was >32 mm, tongue pressure was likely to be increased during THS with greater tongue protrusion.^11^ In the present study, however, PPW‐AP was not correlated with MTP‐L. This discrepancy is unclear, and the relationship between tongue pressure and the PPW‐AP during THS needs to be clarified in future studies. It has been suggested that MTP‐L might be relevant to tongue strength, but might not be directly relevant to the motion of pharyngeal wall.

Oh compared the duration and peak value of sEMG during saliva swallow, THS1 and THS2 and reported both duration and peak value of sEMG increased as tongue protrusion increased (SS < THS1 < THS2).^12^ Fujiwara et al. reported that the duration and integral of sEMG were increased in THS with greater tongue protrusion regardless of the increase/decrease in tongue pressure.^11^ It is suggested that the increased values of sEMG in THS with greater tongue protrusion might be derived by the increased activities of superior hyoid muscles caused by the tongue protrusion and might not necessarily mean increased strength of the tongue or pharynx during THS.^11^ Analysing the length of tongue protrusion during THS only with sEMG is likely insufficient.

The appropriate length of tongue protrusion to elicit greater PPW anterior bulge during THS should be determined by direct observation during VF or CT. In addition, by synchronising high‐resolution manometry with VF or CT, it will be possible to analyse motion and oropharyngeal pressure generated by PPW and BOT which will help clarify the effect of THS on PPW based on the length of tongue protrusion.

## 5 LIMITATIONS

The number of the trials was limited to reduce radiation exposure, and one trial for each swallow does not allow assessment of reproducibility. The second limitation is that scanning required a semi‐reclining posture (45 degree); thus, gravity might influence the motion of the tongue and pharyngeal wall. Future studies exploring these factors by utilising VF would deepen our understanding of THS.

## 6 CONCLUSION

The present study showed that the THS increased the anterior bulge of pharyngeal wall, suggesting that the THS can be used to strengthen oropharyngeal contraction. The degree of tongue protrusion did not linearly correlate with PPW movement during THS. To elicit the greater motion of pharyngeal wall during THS, it is essential to adjust the appropriate length of tongue protrusion based on direct observation of PPW movement.

## CONFLICT OF INTEREST

The authors have stated explicitly that there are no conflict of interests in connection with this article.

## AUTHOR CONTRIBUTIONS

KA and YI made substantial contributions to the study conception and design. KA, YI, DK and HK performed experiments and acquired data. KA, YI, ES and NF analysed and interpreted data. HK, SS and SH performed statistical analysis of data. KA, YI, ES and MG drafted manuscripts. KA, YI, ES, MG, HK, SS and SH edited and revised manuscript. YI and ES approved final version of manuscript.

### PEER REVIEW

The peer review history for this article is available at https://publons.com/publon/10.1111/joor.13246.

## Data Availability

The data sets generated during and/or analysed during the current study are available from the corresponding author on reasonable request.
